# The Extraction of Teeth

**Published:** 1875-12

**Authors:** S. P. Cutler

**Affiliations:** Memphis, Tenn.


### THE EXTRACTION OF TEETH.

BY S. P. CUTLER, M. D., D. D. S., MEMPHIS, TENN.

We are all well aware that this subject has been neglected more than any other branch of our specialty. Why is this so?

Under this head may be included—the causes requiring a resort to the forceps—true and delusive indications; embracing true and false tooth ache, or primary and secondary causes, and other diseased conditions.

The shameful and wholesale slaughter of teeth, by incompetent and often unprincipled men, *dentists so called*, the mischievous career of nitrous oxide gas, now in common use. What constitutes a set of extracting instruments.

In the extraction of the deciduous teeth, nature should be the dentist as a rule, unless she neglects her work. In the permanent tooth, nature having made no provision for their removal, art must come in. All things equal nature removes the deciduous by the absorption of the fangs, their foundation being undermined they must fall out, or rather they are pushed out.

The permanent are not undermined at the bottom, but are worn away and decayed away from the top, the foundation being the last to give away, except in cases of alveolar disease, even then the process is from without inwards.

In all these cases great length of time is generally required with more or less discomfort to the individual. When the deciduous become loose from any cause before the normal, time, and are a source of discomfort, and probable injury to the permanent crowns beneath, then they may be removed prematurely, not otherwise.

When the permanent are being turned from their natural position, the cause should be removed at once. We often see cuspids, and sometimes other deciduous teeth retained beyond puberty and no indications of their successors coming out.

In such cases we are often embarrassed and undecided what to do; even when loosened, we are not always certain a new tooth is coming.

In these cases there is no pathological action only nature's slow coach, stuck in the mud.

Sometimes these teeth make their appearance late in life and may be mistaken for a third dentition.

During the periods of dentition and desquamation, it is well known that there is greater salivary activity than at other times, and a greater tendency to acidity, decay, and systemic predisposition, from incomplete dentification. During this important period, attention should be given to mineral elements in the food.

The subject of filling deciduous teeth is one of great importance, though generally neglected in this country.

The extraction of the permanent teeth is a grave subject; not so much on account of the temporary pain and suffering from the operation, as that of the permanent detriment to the system, which is of momentous significance, as the organism has use for each and every tooth that nature has given. Not a tooth should then be removed, only from an unavoidable necessity, as nature intends all to go to the grave together.

Animals, in their native state, seldom suffer from diseased teeth, they sometimes do when too highly domesticated.

Savages in the forests, sometimes have decayed teeth, though seldom; their habits are more or less artificial. Even the ancient mound builders had decayed teeth, some of which I have now in my possession; and they had means of extracting them also, of which I have evidence. Why is it, that there has been such an increased demand for artificial teeth, since the introduction of anæsthetics? Let us examine.

The idea of suffering pain, from surgical operations of any kind, is a great terror to the mass of mankind, even the slight pain of filling teeth, oftentimes is more ideal than real.

In consequence teeth are constantly suffered to go to decay and destruction, when they could and would be sound.

In the large cities we see gas establishments solely for the extraction of teeth without pain, other establishments where mostly artificial teeth are made, teeth come out by the wholesale, by presence of the GAS, as in the other establishments. Why? Because it *pays best.*

Who are these men as a rule who conduct these establishments. Oftentimes ignorant, unprincipled charlatans.

Many of these are but professional confidence men, no better, if as good, as those infesting drinking saloons and street corners, these last only rob the pockets, while the others not only the pockets, but rob his unsuspecting victim of that which is beyond price, in consequence health impaired and life shortened.

These men have possession of the communities, where they are to a large extent, and have educated them to the belief that plugging teeth is of little value at best, and advises extraction at once, and save money and suffering.

Most of the better class of dentists, some of whom have formerly used gas have now discarded it, in consequence it has fallen into the hands of the class mentioned to a great extent.

These men claim to do cheap dentistry, and in one respect it is cheap, though dearly bought.

Countless numbers of mouths, are being converted from a comparatively physiological to a pathological condition, in consequence the digestive organs, and the entire body to a certain extent, constitutional stamina, lowered, longevity curtailed.

What would an army of soldiers be, who had only false teeth? what would great singers and orators be, with only false teeth? who would wish to marry a young lady with a full set of artificial teeth? though beautiful to behold. These are pertinent questions and apply directly to the case.

If the regimen of children was what it should be, there would be little need of the services of the doctor or dentist. Instead of hot house plants as now, children should be storm and weather proof, like the *oak* and *hickory*, with strong hands and muscles, bones and nerves, able to contend victoriously against the continued conflict, with outside and inside adversities.

These things are chargeable to the dentist, only in part though they have a responsible duty to perform in this direction, by giving suitable instructions to enciente females, in relation to general regimen and habits, and to the young in general.

In relation to saving teeth, instead of extracting, the profession is retrograding; are progressing backwards, and far behind what it was thirty or forty years ago, all other things equal. Why is this so? The answer has been given. There is something lacking somewhere. We might argue as some do, that if we instructed the people how to secure sound teeth, we would have nothing to do, and our occupation, like Othello's would be gone.

Again, we often see teeth lost from alveolar absorption, that could and should be prevented by skillful treatment. We often find one or more loosened teeth from all appearances, too far gone to be saved permanently, and achieve extraction, even when there is a possible chance of arresting the disease by persevering efforts on our part. Mercury is a fruitful cause of loosening teeth, especially in malarial districts where mercury is largely used, many times even where there has been no actual salivation, mercury acts as a remote cause in after years.

Dead teeth in many instances, may be retained for an indefinite time, even after diseased at the root, by proper treatment, in favorable temperaments.

Sometimes it becomes necessary to kill pulps in teeth and fill them, and this is oftentimes done with success, other times failures occur. Some dentists will not run any such risk.

The six year molars are, as a rule, the first permanent teeth that decay, and are often extracted. Some dentists advise their extraction even when sound, instead of the bicuspids, to make room, when too much crowded.

In this I am decidedly opposed, as these are the largest and strongest teeth in the mouth, occupying a side central position, and the most important grinders, being the first of the set having predecessors.

When these teeth are removed early, from the lower jaw, more especially, there is left an ugly depression, allowing the second molars to tilt forwards and the second bicuspids to incline backwards; which sometimes in after life, tends to induce or favor, what is called jimber jaw, or ugly protrusion of chin, making a deformity. These teeth oftener decay than the upper and cause as a rule a worse deformity.

When removeal, from a crowded condition becomes necessary, the greatest amount of judgement is requisite as to what tooth should be removed.

Right here, a profound insight into dental anatomy and histology, is necessary, to a right understanding of the situation.

Permit me to state, that nature puts no unnecessary teeth in the mouth as a rule; it requires all the teeth to serve a man on to extreme old age, in order that he may live out his days in comfort.

The teeth and body should all wear out together, when part of the teeth are removed, the balance have too much work to do, and wear out before their possessor. The teeth should be retained as long as there is any chance of keeping them, as much so as an arm, leg, eye, or any other member of the body. The wisdom teeth, *so called*, are generally under condemnation, before their appearance, are pre-judged, *a priori*, whether guilty of sin or not, and are often disposed of, soon after their appearance. These teeth, which have sinned ever since man got above his ancestral apeship; are often more sinned against than sinning.

These teeth are many times, too lightly disposed of, when they should be saved.

Their position enables them to do more work with the same amount of muscular power, than any other four teeth in the mouth.

These teeth are the last finishing touch nature bestows on man's evolution of the body; here the work is completed; a signal of bodily finish and perfection.

As to extraction of the front teeth, much might be said which space prohibits.

The plan is to save all front teeth where there is any possibility, when only one or two remain, these might be removed to make room for a new set, though there are exceptions even to this rule.

The fangs, if healthy, may be retained and pivoted; when a plate is to be inserted, these may be filled and the plate allowed to rest on them; this is often done. In this way, the contour of the jaw is retained. Thin shells of front teeth, should be filled if possible, and retained.

I used to follow the above plan, and I do now, so far as I can get permission, under favorable circumstances.

Right here, we are greatly in need of some permanent plastic filling, that we have not got; the coming man is to make this discovery and blessings on his head, when he does come.

